# Recognizing difficult trade-offs: values and treatment preferences for end-of-life care in a multi-site survey of adult patients in family practices

**DOI:** 10.1186/s12911-017-0570-x

**Published:** 2017-12-06

**Authors:** Michelle Howard, Nick Bansback, Amy Tan, Doug Klein, Carrie Bernard, Doris Barwich, Peter Dodek, Aman Nijjar, Daren K. Heyland

**Affiliations:** 1 Department of Family Medicine, McMaster University, David Braley Health Sciences Centre, 100 Main Street West, 5th floor, Hamilton, ON L8P 1H6 Canada; 2 School of Public and Population Health at the University of British Columbia, Vancouver, BC Canada; 3 Centre for Health Evaluation and Outcome Sciences, St Paul’s Hospital, Vancouver, BC Canada; 4 Department of Family Medicine, University of Calgary, Calgary, Alberta Canada; 5 Department of Family Medicine, University of Alberta, Edmonton, Alberta Canada; 6 Department of Family and Community Medicine, University of Toronto, Toronto, Canada; 7 Division of Palliative Care, Department of Medicine, University of British Columbia, Vancouver, British Columbia Canada; 8 Division of Critical Care Medicine, St. Paul’s Hospital and University of British Columbia, Vancouver, British Columbia Canada; 9 General Internal Medicine, Faculty of Medicine, St. Paul’s Hospital, University of British Columbia, Vancouver, British Columbia Canada; 10 Department of Critical Care Medicine, Queen’s University, Kingston, Ontario Canada; 11 Clinical Evaluation Research Unit, Kingston General Hospital, Queen’s University, Kingston, Ontario Canada; 12 Department of Public Health Sciences, Queen’s University, Kingston, Ontario Canada

## Abstract

**Background:**

Decisions about care options and the use of life-sustaining treatments should be informed by a person’s values and treatment preferences. The objective of this study was to examine the consistency of ratings of the importance of the values statements and the association between values statement ratings and the patient’s expressed treatment preference.

**Methods:**

We conducted a multi-site survey in 20 family practices. Patients aged 50 and older self-completed a questionnaire assessing the importance of eight values (rated 1 to 10), and indicated their preference for use of life-sustaining treatment (5 options). We compared correlations among values to a priori hypotheses based on whether the value related to prolonging or shortening life, and examined expected relationships between importance of values and the preference option for life-sustaining treatment.

**Results:**

Eight hundred ten patients participated (92% response rate). Of 24 a priori predicted correlations among values statements, 14 were statistically significant but nearly all were negligible in their magnitude and some were in the opposite direction than expected. For example, the correlation between importance of being comfortable and suffering as little as possible and the importance of living as long as possible should have been inversely correlated but was positively correlated (*r* = 0.08, *p* = 0.03). Correlations between importance of values items and preference were negligible, ranging from 0.03 to 0.13.

**Conclusions:**

Patients may not recognize that trade-offs in what is most important may be needed when considering the use of treatments. In the context of preparation for decision-making during serious illness, decision aids that highlight these trade-offs and connect values to preferences more directly may be more helpful than those that do not.

**Electronic supplementary material:**

The online version of this article (doi: 10.1186/s12911-017-0570-x) contains supplementary material, which is available to authorized users.

## Background

When people are faced with the need to make decisions about health care during serious illness or near the end-of-life, they may not be adequately prepared. Most people who are approaching the end of life have a point after which they shift their preferences from a treatment and cure orientation and express a preference for non-invasive treatment aimed at improving quality of life and symptoms [1]. However this may not be clear to health care providers. Treatments given or ordered are often discordant with this preference and may be more intensive than desired [2–4]. Advance care planning (ACP) has been proposed as one solution to improve patient-centred care and ensure that the values and preferences of patients are used to direct health care decisions. ACP is defined as “a process that supports adults at any age or stage of health in understanding and sharing their personal values, life goals, and preferences regarding future medical care” [5]. In prospective and randomized trials, ACP significantly improves outcomes including increased likelihood that clinicians and families understand and comply with a patient’s wishes [6–9], reduces hospitalization at the end of life, results in less intensive treatments at the end of life (according to patients’ wishes) and increases use of hospice services [2, 10].

In some western countries approximately half of adults surveyed in the community have engaged in some aspects of ACP such as thinking about their wishes and who would make treatment decisions on their behalf if they were to become mentally incapable [11, 12], however these activities alone may be inadequate. Advance care planning has been equated with the completion of advance directive documents. However, the consideration and communication of values and preferences in preparation for future decisions made by patients or their substitute decision-makers in consultation with health care providers, are also key aspects of ACP [13]. Furthermore, advance directives have limited utility because they contain instructions that may not apply to the specific situation of the patient when the decision is needed [14] and they do not have legal standing in some jurisdictions.

The success of ACP rests on the assumptions that patients have adequately communicated their values to others who will make specific treatment decisions on their behalf in the future and that those values truly reflect what is most important to the patient at the time of decision-making. However research has shown that substitute decision-makers are often unclear about the patient’s treatment preferences [15], preferences sometimes change over time [16] and patients’ expression of their values can be inconsistent, making them unhelpful as preparation for discussion of treatment decisions with health care providers [17]. A sub-optimal process of clarifying values may contribute to difficulties with ACP. Structured interventions and decision aids have been recommended as tools to improve the process and outcomes of ACP [18–20]. However, a systematic review that examined decision aids for end-of-life decisions found that patient values and treatment goals were not well addressed in the tools, limiting the usefulness of some decisions aids for ACP [21].

Tools for patients such as decision aids are important in ACP because people can construct preferences only when they are faced with choices [22]. Furthermore, consumer theory suggests that patients must recognize trade-offs between features of options so that they can clarify the relative importance, or value, of each [23]. One of the key components of decision-making during serious illness is, for example, the patient’s values regarding maximizing quality versus quantity of life. Well-designed decision aids can support the alignment of decisions with values and preferences of the patient if they help patients recognize trade-offs and link the patient’s values to preferences in a reliable and transparent way [23–25]. If values are not clarified and values and preferences are not clearly linked, a poor decision-making process may arise leading to uncertainty about decisions.

As part of a survey on engagement in ACP among patients in primary care, we asked about values and preferences for life-sustaining treatments using previously published questions and taxonomies [26–28]. The objective of this study was to examine the consistency of ratings of the importance of the values statements and the association between values statement ratings and the patient’s expressed treatment preference, to understand whether and how these values statements could be useful in guiding deliberations with health care providers about use of life-sustaining treatments.

## Methods

### Setting and design

We conducted a cross-sectional study in primary care practices between October 2014 and March 2015 as part of a larger project to improve the quantity and quality of ACP discussions in primary care practices. The methods of the main study that provided these data have been described previously [29]. The study was conducted in a convenience sample of 20 family physicians’ practices in Canada: 13 practices in the province of Ontario, five in Alberta, and two in British Columbia. The family practices were located in the same communities as members of the research team and were invited by physician members of the research team. Included practices had a defined patient population (versus episodic walk-in clinics) and provided comprehensive family medicine services (as opposed to a focused practices such as psychotherapy or sports medicine). A variety of practice types were intentionally invited. All were located in urban centres, three were teaching clinics and 17 were community-based private practices, 17 employed allied health professionals, and 17 were practices with more than one physician in the same office.

### Participants

Staff or clinicians in the family practices invited consecutive eligible patients to speak to the research assistant. Eligible patients were 50 years of age and older, could read and speak/understand English, and did not have cognitive impairment that would limit participation. The referring staff member or clinician knew the age of the patient and determined whether the patient was cognitively suitable but a formal assessment of cognitive impairment was not done. The research assistant met with the patients in a private space in the clinic and explained the study and obtained informed consent.

### Questionnaire

We adapted a questionnaire that was previously developed and validated for use in hospitalized patients [27]. The hospital version has face and content validity and good ratings of clarity and low emotional burden [30]. We modified this questionnaire for use in primary care and piloted it with 25 patients in primary care to assess its clarity, sensibility, and acceptability. Revisions were made to improve clarity. These questions addressed the patient’s engagement in ACP (see Additional file 1).

Our definitions of values and preferences are taken from a consensus-based framework on end-of-life communication and decision-making [31]. ‘Values’ refer to an expression of a person’s overarching philosophies or most important priorities in life (such as maximizing quality of life or time spent with family). Preferences refer to specific preferred options for treatments (such as use of resuscitative treatments or not) or preferred health states (conditions that would be acceptable or unacceptable) [17]. To assess the values of respondents, we used the End of Life (EOL) Values Scale [26], a multi-item questionnaire that asks the respondent to rate the level of importance (1 = not important;10 = extremely important) of each item in a list of common values that are relevant to EOL care (Table 3). The values questionnaire was based on a previous studies on EOL values in community dwelling elderly individuals [26, 28], and adapted through expert input and pilot testing with seriously ill patients in hospital [15] and in primary care [29]. To elicit preferences for use of life-sustaining treatments, we used a taxonomy of different levels of the use or non-use of life-sustaining treatments (shown in Table 2). This taxonomy was developed with inputs from medical experts and has been used extensively in our previous research [27, 32]. The questionnaire also included socio-demographic items and the brief clinical frailty scale with text description and pictographs on which patients were asked to rate themselves [33]. Patients self-completed the questionnaire.

### Sample size and statistical analysis

A sample size of 30-50 patients was targeted in each family practice to attempt to obtain a representative sample for each practice, because the overall study was designed to provide baseline data to each practice for the purpose of quality improvement.

Characteristics of patients were described as counts and percentages for categorical variables and as means with standard deviations and ranges for continuous variables. Median and inter-quartile ranges of the ratings on the EOL Values Scale were reported because the data were skewed.

To assess the internal consistency of respondents’ stated values, we calculated the Pearson correlation coefficients among the various value statements in the EOL values scale. Most items in the EOL Values Scale are associated with either prolongation or shortening of life. We used the same a priori hypotheses regarding which values would be correlated and in which direction as in a previous paper that used the same rating scales [17]. In that paper the hypotheses were formulated by the authors based on whether the value statement related to prolonging or shortening of life. For example two values statements that related to prolonging life such as wanting to live as long as possible and the belief that life should be preserved were expected to be strongly positively correlated. The survey in the current study included an additional value statement about hospital avoidance and two authors (MH, DKH) constructed the a priori hypotheses for this question.

Similar to the values statements wherein a high rating of importance suggests a desire for either prolongation or shortening of life, the treatment preference items were oriented to either prolongation or shortening of life. We therefore hypothesized that higher importance rating of values aligned to life prolongation would be associated with treatment preferences involving life-prolonging measures (beyond comfort care only), and vice versa. Correlations were described in terms of size using the rule of thumb of Hinkle (0.9-1.0 = very high; 0.7-0.9 = high; 0.5-0.7 = moderate; 0.3-0.5 = low; 0.0-0.3 = negligible) [34]. For the analysis of association between values and preferences we first used the five categories of response related to preferences; then, to simplify the options, we repeated the analysis after collapsing these responses to three categories: full measures including cardiopulmonary resuscitation (CPR), machines or medical treatments but not CPR, and comfort care only. Patients were excluded from analysis if they answered ‘unsure’ or did not respond to the item. Box plots were used to illustrate the distribution of the importance of stated values by the preference option for life-sustaining treatment chosen. The concordance between respondents’ stated values and their chosen preference for use of life sustaining treatments was measured by Kendall’s τ-b statistic. Kendall’s τ-b is the excess concordance (C-(1-C)) where C is the probability that an individual who puts a higher importance on a given value related to prolonging life than someone who put a lower importance on the same value. Kendall’s τ-b ranges from 1 to −1 where 1 indicates perfect concordance, −1 indicates perfect discordance and 0 indicates no association. We first calculated the statistic using the five categories of preference, and based on visual inspection of the boxplots, also calculated with three categories of preference where the middle categories between ‘all possible measures including CPR’ and ‘comfort care only’ (the extreme points) were collapsed to a single category.

#### Ethics and consent

Ethics approval for the study was obtained from the Research Ethics Board of each participating institution (Hamilton Integrated Research Ethics Board [14-582], Queen’s University Health Sciences and Affiliated Teaching Hospitals Research Ethics Board [6013436], University of British Columbia Office of Research Ethics [H14-02836], University of Alberta Health Research Ethics Board [Pro00051082]). Verbal informed consent was obtained from participants.

## Results

The survey was completed by 810 patients (92% completion rate among patients approached by family practice staff). The number of patients recruited per family practice ranged from 23 to 140 (in one multi-physician practice approximately 20 patients per physician were recruited). The mean age of patients was 66 years (range 50-95 years) and 56% were female (449/809). Most patients were Caucasian (88%; 713/810). The median score on the clinical frailty scale was 2 (corresponding to ‘well’).

There was a ceiling effect, that is the median rating was 10 out of 10 for three values statements: being comfortable and suffering as little as possible, having more time with family, and that death is not prolonged. The lowest rated statements were for the belief that life should be preserved and avoiding hospitalization (median rating = 6) (Table 1). Across all items 11% to 13% of the statements were answered as ‘unsure’.

**Table 1:** Rating of importance of each issue in thinking about the kinds of medical treatments wanted or not wanted near the end of life

Item: How important …	median(q1,q3) (n)	Unsure	Missing or declined % (n/N)
I be comfortable and suffer as little as possible?	10(9,10) (793)	13% (100/793)	2% (15/793)
I have more time with my family?	10(8,10) (785)	13% (102/785)	2% (15/785)
I live as long as possible?	8(5,10) (777)	12% (97/777)	2% (15/777)
I avoid being attached to machines and tubes?	9(7,10) (753)	11% (83/753)	2% (14/753)
My death is not prolonged?	10(8,10) (750)	11% (81/750)	2% (14/750)
Belief that nature should be allowed to take its course?	8(5,10) (760)	12% (89/760)	2% (14/760)
Belief that life should be preserved?	6(3,8) (736)	11% (83/736)	2% (13/736)
I respect the wishes of other family members?	7(4,10) (778)	12% (97/778)	2% (14/778)
I avoid hospitalization?	6(3,9) (762)	12% (90/762)	2% (13/762)

Of the 24 a priori predicted correlations between importance ratings for value statements, 14 were statistically significant in the expected direction but 11 of those were negligible correlations (defined as *r* < 0.3) (Table 2). For example, we hypothesized that there would be a strongly positive correlation between the importance of being comfortable and suffering as little as possible and the importance of avoiding machines, and between the importance of the belief that death should not be prolonged and the importance of the belief that nature be allowed to take its course. However, the correlations were negligible (0.24, 0.19 respectively). Of the 12 correlations we expected to be negative, four were in fact significantly positive; for example there was a positive correlation between the importance of living as long as possible and being comfortable and suffering as little as possible (*r* = 0.08, *p* = 0.036). We hypothesized that the correlation between the importance of avoiding tubes and machines and the belief that life should be preserved would be strongly negative, and found that it was negligible (−0.03, *p* = 0.494). Similarly, we expected the correlation between the importance of living as long as possible and avoiding machines to be strongly negative and observed no significant results (*r* = 0.02, *p* = 0.518) The largest of any correlation was (moderate): the correlation between living as long as possible and having more time with family (*r* = 0.64, *p* < 0.001).

**Table 2:** Correlations between different values pertaining to decisions about life-sustaining treatments

	a	b	c	d	e	f	g	h	i
a) How important is it that I be comfortable and suffer as little as possible? (shorten)	1.00	0.11 0.0022	0.08*↓* 0.0364	0.24**↑** <.0001	0.28**↑** <.0001	0.09**↑** 0.0130	−0.04*↓* 0.2882	0.05 0.1453	0.12 0.0011
b) How important is it that I have more time with my family? (prolong)		1.00	0.64**↑** <.0001	−0.03*↓* 0.3449	−0.08*↓* 0.0381	0.07 0.0521	0.37 <.0001	0.43 <.0001	0.09↓ 0.0129
c) How important is it that I live as long as possible? (prolong)			1.00	0.02*↓* 0.5183	−0.12*↓* 0.0009	0.10*↓* 0.0066	0.52**↑** <.0001	0.43 <.0001	0.13↓ 0.0003
d) How important is it that I avoid being attached to machines and tubes? (shorten)				1.00	0.46**↑** <.0001	0.16**↑** <.0001	−0.03*↓* 0.4943	0.02 0.5562	0.29**↑** <.0001
e) How important is it that my death is not prolonged? (shorten)					1.00	0.19**↑** <.0001	−0.14*↓* 0.0002	0.03 0.4868	0.19**↑** <.0001
f) How important is a belief that nature should be allowed to take its course? (shorten)						1.00	0.18 <.0001	0.15*↓* <.0001	0.14**↑** 0.0002
g) How important is the belief that life should be preserved? (prolong)							1.00	0.38 <.0001	0.04↓ 0.3300
h) How important is it that I respect the wishes of other family members regarding my care? (neutral)								1.00	0.17 <.0001
i) How important is it that I avoid hospitalization? (neutral)									1.00

Association between value statements and likely influence on length of life indicated in brackets (prolong, shorten, neutral)

A priori, correlations indicated ↑ were expected to be strongly positive and correlations indicated ↓ were expected to be strongly negative

*P* values are written under correlation coefficient

Most patients preferred the treatment option that excluded CPR (92%). Nearly one-third of patients preferred ‘comfort measures only’ (30%), 7% preferred full medical care without resuscitation or breathing machines, and 36% preferred short term use of machines but not CPR, switching to comfort measures or all possible measures but without CPR (4%). Only 8% preferred all possible measures including CPR, and 15% were unsure or did not answer the question (Table 3).

**Table 3:** Preferences for care if life supports were needed to stay alive

Response item	% (n)
All possible measures including resuscitation (CPR)	8 (65)
All possible measures, no resuscitation.	4 (35)
Machines only in the short term, change focus to comfort measures, no resuscitation (CPR) or breathing machines.	36 (294)
Use full medical care, no resuscitation (CPR) or breathing machines	7 (55)
Comfort measures only	30 (239)
Unsure	13 (106)
Missing or declined	2 (16)

*CPR* cardiopulmonary resuscitation

The Kendall’s τ-b for the correlation between the importance rating for values and the five-option preference question (1 = all measures including CPR, 5 = comfort care only, no CPR; increasing number means less use of life sustaining treatments) were negligible, ranging from 0.03 to 0.13. The highest correlations between values and preferences were found for ‘nature allowed to take its course’, ‘death not prolonged’, and ‘life should be preserved’ (Kendall’s τ-b = 0.12, 0.13 and −0.12 respectively, all statistically significant). These correlations were in the expected direction where a higher importance rating of values related to not prolonging life correlated positively with the choice on the preference scale and values associated with prolonging life correlated negatively with the preference item.

**Fig. 1:**
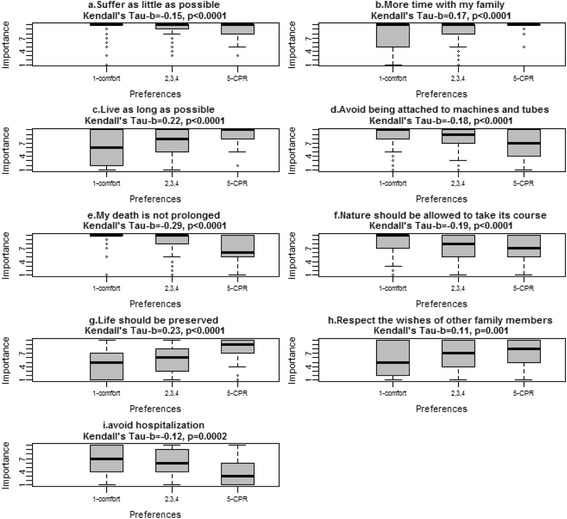
Box-plots of relationship between importance ratings for values and preference option for life-sustaining treatment, CPR – cardiopulmonary resuscitation. 1 = Use comfort measures only with a focus on improving my quality of life and comfort. Allow natural death and no artificial prolongation of life and no resuscitation. 2, 3, 4 = combined categories of: Use full medical care to prolong my life but if my heart or my breathing stops, no resuscitation (CPR) or breathing machine; Use machines only in the short term to see if I will get better but if my illness is prolonged, change focus to comfort measures only. If my heart stops, no resuscitation (CPR); Use machines and all possible measures with a focus on keeping me alive but if my heart stops, no resuscitation. 5 = Use machines and all possible measures including resuscitation (CPR) with a focus on keeping me alive at all costs

On visual inspection of the figures, the mean importance ratings of values statements discriminated better between the preference for ‘all possible measures including CPR’ and ‘comfort care only’ (the extreme points) but there was less variation across the other three options for care. After collapsing to three categories for the preference question: all measures including CPR, collapsing the options for life-prolonging treatments without CPR, and comfort care only, the correlations increased but remained negligible, ranging from 0.11 to 0.29 (Fig. 1).

## Discussion

In this multi-site cross-sectional survey of approximately 800 adult patients in the family practice setting, patients’ ratings of a set of values statements and response to a question about treatment preference demonstrated that there is a ceiling effect for some values statements, the ratings of some values statements are inconsistent or illogical when compared to each other, and the ratings given to the importance of some values statements are minimally associated with the expected treatment preference. Taken together, these findings suggest that patients are not able to discriminate values that compete with or trade-off with each other and have trouble linking their values statements to preferences for medical treatments.

Patient decision aids have been proposed as a way to improve decision-making relating to serious illness or end of life [20, 35–37] and values clarification is a central component of these decision aids [24]. Our results suggest that patients do not recognize that trade-offs may be required with respect to receiving health care that would be consistent with values. For example if trade-offs are recognized we would expect a negative correlation between the importance ratings of living as long as possible and being as comfortable and suffering as little as possible. However both items had a median importance rating of 10 out of 10, and the correlation between them was weak. There is growing literature supporting the need for approaches to designing questions such as ranking, constant sum, and discrete choice experiments that ask patients to specifically compare the relative importance of each issue [38, 39]. It is possible that asking patients to consider the importance of each issue on its own, without considering any trade-offs, did not necessarily elicit informed values from individuals. The decision theory literature suggests values only become informed and stable when individuals have the opportunity to trade off one aspect with another and reflect on those trade-offs [40, 41]. Values can then be determined by considering the amount one is willing to sacrifice from something else [42]. There appears a role for these questions to not just elicit, but also help clarify patients’ values, and help them recognize the trade-offs in end of life care, Understanding how a patient prioritizes his/her values, may then allow the clinician to guide the patient to further *prioritize* decision options if they are faced with decisions whose outcomes will not align with all of their values. Further research should explore whether these explicit questions can overcome the problems of ceiling effects and inconsistency among values ratings that arose with our results.

We used a taxonomy for preferences regarding life-sustaining treatments that provided five options, three of which were for various degrees of medical care without CPR that would attempt to prolong life to some extent. The patterns of values statement ratings across these three medical care options suggested that patients may not have understood the differences between the nuanced treatment approaches because the correlations with values statements were very low and improved when the taxonomy was collapsed to combine these three categories. For patients to express a preference for a treatment approach and appreciate the connection to their values, attention to language and level of detail regarding the goals of the treatment approaches are needed.

The findings of this study could be helpful in designing decision aids that elicit the values that are most strongly associated with treatment preferences and therefore support the process of ACP because they are informative to patients, their substitute decision-makers and clinicians. For example, our results have demonstrated that some values in our values questionnaire such as being comfortable and suffering as little as possible, and having more time with family would not be helpful to elicit in a clinical setting because of a ceiling effect in importance ratings. The values statements that might have utility were the importance of living as long as possible, the belief that death not be prolonged and the belief that nature be allowed to take its course or that life should be preserved, because these were the items with the greatest association with preferences for life sustaining treatments. In this study, values and preferences were assessed at one point in time and the question of the usefulness of considering them in advance arises. Studies have reported that some patients change their preferences once they experience a health condition [16, 43], and some have difficulty predicting how they will feel about future health states [44]. Thus, it is important that the patient’s values and preferences be revisited with their clinicians on an ongoing basis, and in particular if health deteriorates. Prior values clarification exercises still have utility because the process may help patients to be informed and more able to confront trade-offs so they can arrive at better decisions than if they are thinking about these issues for the first time, or the patient may lose capacity making it necessary to rely on past expressions of values. Preferences for health care options can remain stable even with changing experience of health care [45], and the majority of patients have stable preferences relating to end of life decisions, especially those who have completed an advance directive [16].

Our finding that 13% of patients are uncertain about their preferences regarding use of life-sustaining treatments may not be surprising given that the sample of generally healthy patients with an average age of 66 may not have given much prior consideration to these issues. However, similar findings have been reported in seriously ill older adults in hospital who might be expected to have considered decisions about life-sustaining treatments [17]. Similar to other findings in older adults who live in the community [7], only 8% of patients in the current study expressed a preference to receive CPR, It may be that patients do not understand the implications of their preference regarding CPR [46], therefore there is an imperative to ensure high quality and timely communication and decision-making. Guideline-recommended elements of goals of care discussions that are desired by patients in hospital, such as discussions about values happen infrequently [3].

A strength of this study is that we surveyed patients in multiple family practices across regions. It is a limitation that the values statements instrument we used was not designed for the purpose of this study- to assess consistence of values and compare values to treatment preferences A more constrained technique with ranking of values may increase the recognition of trade-offs. It is possible that in this population of patients from primary care settings aged 66 years on average and who were not acutely ill, the responses to questions that are relevant to decisions during serious or critical illness may differ from responses in the future when faced with the situation. In addition, family practices and patients were not randomly sampled and we cannot be certain of the generalizability of the study results. Finally, our participants were predominantly Caucasian and had a higher level of education; results may not apply to people who are not Caucasian or who have lower educational attainment.

## Conclusion

The reported values and preferences related to use of life-sustaining treatments among primary care patients reflect considerable inconsistency and discordance. To engage in high quality ACP, patients will need assistance to recognize possible trade-offs among their values and to understand the relationship between their values and their treatment preferences. The results demonstrate a need to improve the processes and tools to support advance care planning. These efforts should specifically include approaches that recognize trade-offs among values and link values explicitly to treatment preferences.

## Additional file

Additional file 1: Questionnaire asking patients about engagement in advance care planning. (DOCX 37 kb)

## Abbreviations

ACP: Advance care planning
CPR: Cardiopulmonary resuscitation
EOL: End of life
