# Supplementary material for: Recognizing difficult trade-offs: values and treatment preferences for end-of-life care in a multi-site survey of adult patients in family practices
Source: BMC Med Inform Decis Mak. 2017 Dec 6;17:164. doi: 10.1186/s12911-017-0570-x (PMC5719577; doi:10.1186/s12911-017-0570-x)
Supplement: Additional file 1: — Questionnaire asking patients about engagement in advance care planning. (DOCX 37 kb) [file 12911_2017_570_MOESM1_ESM.docx]

**Audit of Communication, CarE Planning, and DocumenTation**

**The ACCEPT Study**

#

**For the Family Practice Patient**

**Date**

___-_____-_______

DD MMM YYYY

You are being asked to participate in a questionnaire. We are looking to understand your thoughts regarding the kinds of medical treatments that you’d like to receive in the event your physical health deteriorated. This questionnaire should take no more than 10 minutes of your time to complete. Participation in this questionnaire is completely voluntary. The answers to these questions will help us understand how we can improve the quality of patients’ medical care in the event of a serious illness. Please read the questions carefully and follow the instructions to provide your answers.

**There are no right or wrong answers. Your answers will not impact the care you will receive from your family physician or from the hospital in the event you are admitted**.

Completely honest answers are most helpful!

If you do not wish to complete this questionnaire, please return the questionnaire to the receptionist.

If you have already completed this questionnaire at a previous appointment please do not complete it again. Kindly return the blank questionnaire to the receptionist.

**If you have any questions or concerns about the study, please feel free to contact:**

**If you have questions about advance care planning, please contact your family physician for more information.**

#### Section 1: Decisions About Your Health Care

1. **Have you heard about Advance Care Planning?**

❑ Yes ❑ No

***Advance Care Planning*** is thinking about your future health care treatment decisions and what your wishes are for end of life care.  It is also about talking with your close family, friends, and health care providers (like your doctor) so they know your thoughts and wishes if you are not able to speak and make decisions yourself.  It also involves naming someone to make medical decisions for you if you are not able to speak for yourself.

1. **Have you ever thought about what kinds of medical treatments you would want, or not want, if you were to get very sick and be in a hospital? By medical treatments we mean** the use of cardiopulmonary resuscitation (CPR), breathing machines, dialysis, artificial nutrition, Intensive Care Unit (ICU) admission, etc.

❑ Yes ❑ No

1. **Think about *if* you were to get a serious, life-threatening illness at some time in your life. Please rate the importance of each issue below in thinking about the kinds of medical treatments you would or would not want**

| Please circle one answer for each question on a scale of 1 to 10 where 1 in ‘not at all important’ and 10 is ‘very important.’ | | | | | | | | | | | | | |
| --- | --- | --- | --- | --- | --- | --- | --- | --- | --- | --- | --- | --- | --- |
| a) How important is it that I **be** **comfortable and suffer as little as possible?** | **Not at all important** | 1 | 2 | 3 | 4 | 5 | 6 | 7 | 8 | 9 | 10 | **Very**  **important** | **Unsure or Undecided** |
| b) How important is it that I **have more time with my family?** | **Not at all important** | 1 | 2 | 3 | 4 | 5 | 6 | 7 | 8 | 9 | 10 | **Very**  **important** | **Unsure or Undecided** |
| c) How important is it that I **live as long as possible?** | **Not at all important** | 1 | 2 | 3 | 4 | 5 | 6 | 7 | 8 | 9 | 10 | **Very**  **important** | **Unsure or Undecided** |
| d) How important is it that I **avoid being attached to machines and tubes?** | **Not at all important** | 1 | 2 | 3 | 4 | 5 | 6 | 7 | 8 | 9 | 10 | **Very**  **important** | **Unsure or Undecided** |
| e) How important is it that my **death is not prolonged?** | **Not at all important** | 1 | 2 | 3 | 4 | 5 | 6 | 7 | 8 | 9 | 10 | **Very important** | **Unsure or Undecided** |
| f) How important is a **belief that nature should be allowed to take its course?** | **Not at all important** | 1 | 2 | 3 | 4 | 5 | 6 | 7 | 8 | 9 | 10 | **Very**  **important** | **Unsure or Undecided** |
| g) How important is the **belief that life should be preserved?** | **Not at all important** | 1 | 2 | 3 | 4 | 5 | 6 | 7 | 8 | 9 | 10 | **Very**  **important** | **Unsure or Undecided** |
| h) How important is it **that I respect the wishes of other family members regarding my care?** | **Not at all important** | 1 | 2 | 3 | 4 | 5 | 6 | 7 | 8 | 9 | 10 | **Very**  **important** | **Unsure or Undecided** |
| i) How important is it **that I avoid hospitalization?** | **Not at all important** | 1 | 2 | 3 | 4 | 5 | 6 | 7 | 8 | 9 | 10 | **Very**  **important** | **Unsure or Undecided** |

**4. a) Have you talked with anyone about what medical treatments you would want or not want at the end of life?**

❑ Yes (answer b and c) ❑ No (answer d)

**4. b) If YES, with whom?** Check (√) all that apply

- Family Doctor
- Other Doctor
- Nurse
- Social Worker
- Spiritual Care Worker
- Family Member(s)
- Surrogate Decision Maker
- Lawyer
- Other (specify): _________________________

**4. c) Who brought up the discussion?**

- I brought it up
- A doctor brought it up
- A family member brought it up
- A lawyer brought it up
- Other (specify): _____________________________________________

**4. d) If NO, why haven’t you talked with someone?**

- Did not know about advance care planning
- Did not see this as necessary
- I tend to leave medical decisions to my physician
- I think my family will know what to do
- Other (specify): _____________________________________________

1. **How comfortable are you talking to your family doctor about medical treatment options concerning the end of your life?**
   - Very Uncomfortable
   - Quite Uncomfortable
   - Neutral (neither uncomfortable nor comfortable)
   - Quite Comfortable
   - Very Comfortable
2. **What is the one thing that makes it very hard for you to talk to your family doctor about medical treatments at the end of life?**

_____________________________________________________________________

_____________________________________________________________________

_____________________________________________________________________

_____________________________________________________________________

_____________________________________________________________________

_____________________________________________________________________

1. **How comfortable are you talking to your family members about medical treatment options concerning the end of your life?**
   - Very Uncomfortable
   - Quite Uncomfortable
   - Neutral (neither uncomfortable nor comfortable)
   - Quite Comfortable
   - Very Comfortable
2. **What is the one thing that makes it very hard for you to talk to your family members about medical treatments at the end of life?**

_____________________________________________________________________

_____________________________________________________________________

_____________________________________________________________________

_____________________________________________________________________

_____________________________________________________________________

_____________________________________________________________________

**Many people have gone to a lawyer and completed a power of attorney for financial and property matters, or a last will and testament. The following questions pertain to planning you have done as it relates to your future health care only and not financial matters.**

**9. a) Have you written down your wishes about the medical treatments you would want (or not want) in the event you are unable to speak for yourself? (For example, do you have an advance directive or living will or another written document?)**

❑ Yes ❑ No ❑ Unsure

**9. b) Have you named someone, in writing, to be your substitute decision maker for medical treatment decisions? (eg. Power of Attorney for Person Care, Personal Directive, Representation Agreement)**

❑ Yes ❑ No

1. **At this point in time, if life supports were needed to keep you alive, which option would you prefer for your care?** Please check (√) one.

| ❑ | Use machines and all possible measures including resuscitation (CPR) with a focus on keeping me alive at all costs. |
| --- | --- |
| ❑ | Use machines and all possible measures with a focus on keeping me alive but if their heart stops, no resuscitation. |
| ❑ | Use machines only in the short term to see if I will get better but if the illness is prolonged, change focus to comfort measures only. If my heart stops, no resuscitation (CPR). |
| ❑ | Use full medical care to prolong my life but if my heart or my breathing stops, no resuscitation (CPR) or breathing machines. |
| ❑ | Use comfort measures only with a focus on improving my quality of life and comfort. Allow natural death and no artificial prolongation of life and no resuscitation. |
| ❑ | Unsure |
